# Everyday Cyborgs: On Integrated Persons and Integrated Goods

**DOI:** 10.1093/medlaw/fwy003

**Published:** 2018-02-22

**Authors:** Muireann Quigley, Semande Ayihongbe

**Affiliations:** 1 Birmingham Law School, University of Birmingham, Edgbaston, B15 2TT, UK; 2 Newcastle Law School, Newcastle University, 21-24 Windsor Terrace, Newcastle, NE1 7RU, UK

**Keywords:** Medical devices and prostheses, Law’s boundary-work, Data and privacy, Biohacking, Law, regulation, and technology, Intellectual property rights

## Abstract

Using the metaphor and actuality of the ‘everyday cyborg’, this article makes the case that the law is ill-equipped to deal with challenges raised by the linking of the organic, biological person with synthetic, inorganic parts and devices. For instance, should internal medical devices that keep the person alive be viewed as part of the person or mere objects (or something else)? Is damage to neuro-prostheses (eg nervous system integrated limb prostheses) personal injury or damage to property? Who ought to control/own the software in implanted medical devices? And how should the law deal with risks around third-party device access (including that of unauthorised access and hacking)? We argue that satisfactorily answering such questions will likely require a re-analysis of the conceptual and philosophical underpinnings of the law, as well as the law itself. To demonstrate this, we examine the uncharted terrain which everyday cyborgs pose for the law, looking in particular at five areas: (i) medical device regulation, safety, and product liability; (ii) damage to devices and liability; (iii) data and privacy; (iv) security and biohacking; and (v) intellectual property rights. The article highlights how advancing biotechnology continues to reveal, and prompts us to confront, lacunae within the law. Our analysis calls particular attention to law’s boundary-work (how the law utilises and incorporates supposed ontological and moral boundaries) and the challenges which everyday cyborgs pose to this.

## I. INTRODUCTION

Everyday cyborgs are all around us. They are persons with replacements and augmentations ranging from the simple to the extraordinarily complex, for example, artificial joint replacements, implanted devices such as pacemakers and the total artificial heart, and limb prostheses. An increasing number of people in the UK and worldwide rely on such medical devices to monitor their physiological function, deliver medication, supplement the functioning of a body part, or replace missing body parts.^1^ Some of these support a person’s bodily function. Others are aimed at restoring the body to its normal function. Some may even enhance such functioning.^2^ In this article, we contend that the law is ill-equipped to deal with questions and challenges raised by the linking of the organic, biological person with synthetic, inorganic parts and devices. For instance, should internal medical devices which keep the person alive be viewed as part of the person or mere objects (or something else)? Is damage to neuro-prostheses (e.g. nervous system integrated limb prostheses) personal injury or damage to property? Who ought to control/own the software in implanted medical devices? And how should the law deal with risks around third-party device access (including that of unauthorised access and hacking)? Our aim is not to give fulsome answers to such complex questions, but to begin to analyse the uncharted terrain that everyday cyborgs pose for the law. As such, this article represents the first sustained analysis of the challenges wrought by everyday cyborgs across multiple areas of the law. In so doing, it also reveals some of the problematic conceptual apparatus, which sits at the very heart of law’s structure. The consequence of this, we will conclude, is that satisfactorily answering questions such as those just set out will likely require a re-analysis of the conceptual and philosophical underpinnings of the law, as well as the law itself.

In order to do this, in Section II, we discuss the idea of the everyday cyborg, outlining some of the technologies of the everyday cyborg and setting out in broad terms the nature of the challenges they pose. In Section III, we identify and examine some specific legal issues, focusing on five areas: (i) medical device regulation, safety, and product liability; (ii) damage to devices and liability; (iii) data and privacy; (iv) security and biohacking; and (v) intellectual property rights. This section highlights how advancing biotechnology continues to reveal, and prompts us to confront, lacunae within the law. Following that, in Section IV, we examine some of the conceptual and normative implications identified in the previous section. In so doing, we call attention to law’s boundary-work; that is, how the law utilises and incorporates supposed ontological and moral boundaries, something which influences not only its approach to specific problems, but also its underlying structure.

We will see throughout this article that everyday cyborgs and their technologies are encompassed by diverse and indeed disparate areas of law. Many of the difficulties, challenges, and gaps we identify have been given little explicit legal consideration up until now. And even where the issues discussed shade into existing debate and legal provisions (e.g. data protection and privacy), the era of smart prostheses and implanted devices gives rise to novel dimensions. These arise, in particular, because of the assemblage of integrated persons and integrated goods. As will become apparent, everyday cyborgs are *integrated persons*; that is, the integration of the biological person with the technological. Any conceptual or legal difficulties in this respect are compounded by the nature of the technologies themselves. Increasingly, these are what can be called *integrated goods*; that is, devices which not only have a physical existence, but which are also capable of running software and of collecting, analysing, and transmitting data. Although integrated goods themselves are not a new thing (all computers, e.g. are such goods), the assemblage of integrated persons and integrated goods generates a level of conceptual and normative complexity that the law has not hitherto had to deal with head on. This makes deeper analysis an imperative. Using the metaphor and actuality of the ‘everyday cyborg’, this article takes an important step in that direction.

## II. TECHNOLOGIES OF THE EVERYDAY CYBORG

Gill Haddow and colleagues view everyday cyborgs as persons who are ‘hybrid[s] of machine and organism living in modern society’.^3^ They are ‘everyday’ cyborgs as distinct from the familiar and more extraordinary imagery of science fiction; for example, Asimov’s Bicentennial Man or Seven of Nine and Locutus of the Borg from Star Trek. They are ordinary persons with implantable medical devices. The image of the everyday cyborg echoes Haraway’s famous ‘cyborg manifesto’ in which she describes a cyborg as being ‘a creature of social reality as well as a creature of fiction’.^4^ Everyday cyborgs are where the social reality of the present meets the science fiction of the future, embodying—as they do—ever increasingly sophisticated technologies. As such, Haddow and colleagues are mainly interested in persons with ‘smart’ technologies and not ‘implantable technologies of the “carpentry kind” such as hip or knee joints, or other static prosthetics’.^5^ Their focus is on technologies, which broadly correspond to the original meaning of the word ‘cyborg’ as ‘cybernetic organism’.^6^ As such, they conceptualise everyday cyborgs as persons with implantable technologies that involve a high degree of automation.^7^ In this vein, much of Haddow’s work on everyday cyborgs focuses on people with internal cardioverter defibrillators (ICDs). These are devices somewhat like pacemakers which monitor and regulate a person’s cardiac rhythm, but can also deliver an electric shock should the heart’s electrical activity and rhythm become disordered. No intervention from the person or third parties is needed.

It will become obvious that the weight of our interest also lies in the direction of so-called smart technologies. Notwithstanding, Thomas’ recent observation that there is a lack of consistency on the use of this term,^8^ and Haddow and colleagues’ conclusion that ‘smart is a complex concept that holds multiple meanings’,^9^ we take there to be a large overlap between smart and integrated goods. Thus, integrated goods are ‘smart’ in the sense that they have software which executes algorithms enabling functionalities such as data collection, analysis, and transmission.^10^ Indeed, it is exactly because of the increasing sophistication and smartness of devices that a number of the challenges we identify arise. Nevertheless, whilst we draw on Haddow and colleagues’ conceptualisation, we also extend it. For us, everyday cyborgs are (i) persons with ‘technologies of the carpentry kind’ such as artificial joints, as well as simple prosthetics such as aesthetic arm prostheses and cosmetic ocular prosthetics (artificial eye); (ii) those carrying devices, such as insulin pumps, which are automated to monitor blood glucose and deliver insulin; (iii) persons with implanted medical devices such as pacemakers, ICDs, cochlear implants, or deep brain neurostimulators, which regulate or replace some physiological function or other; and (iv) persons with complex prosthetics such as retinal prostheses (‘the bionic eye’) or myoelectric prosthetic arms.^11^

This wider conceptualisation has analytical benefits. By looking more widely than strictly smart or integrated, we encompass a much broader range of technologies. This allows us to consider them on a spectrum from simpler, passive, and less technologically sophisticated devices to more active, advanced, and complex ones. It is imperative that this is done because the parts and devices at issue are vast in number and diverse in function (of which those mentioned in this article are merely representative). The technologies of the everyday cyborg range from the simple to the complex, from the well established to the cutting edge. The sheer volume and variety of the devices make any attempt at classification not only difficult, but risks being overly reductive. For instance, Burleson and Carrara suggest that ‘a taxonomy of [implanted medical devices] can be defined by several dimensions’.^12^ These include physical location, the device’s sensing functions, computational capabilities, data storage, communication modes and capabilities, energy requirements, and security vulnerabilities. Even a taxonomy constructed along such seemingly comprehensive lines would neither capture all the technologies of the everyday cyborg nor the significance of their different aspects for the law. For example, it misses the significance of functionality construed not simply in terms of device functionality, but the functionality of and to persons. Similarly, it does not capture the level of integration with or dependency of persons in relation to different devices.

This article casts the technological and conceptual net wide to include a broad spectrum of technologies. Doing this permits us to more fully explore the challenges for the law which different technologies and different modes of integration with persons might give rise to. What we will see is that, in general, challenges arise because of (i) the transgression of the bodily boundary, (ii) the integration of the technologies with persons, (iii) the linking of the biological with synthetic materialities, and (iv) the nature of the technologies as integrated goods with both physical and software components. These dimensions begin to emerge as we examine some of the key legal dimensions of everyday cyborg technologies in the next section, and will be more explicitly discussed in Section IV, where we will examine how they relate to law’s boundary-work.

## III. IDENTIFYING THE LEGAL ISSUES

### A. Medical Device Regulation, Safety, and Product Liability

Recently Laurie has noted that the law often ‘creates artificial constructs that become the object of regulatory attention of dedicated regulators who operate within legally defined spheres of influence or “silos”’.^13^ What we get is a ‘taxonomy of regulated “objects” such as “data”, “tissue”, “embryos”, “devices”, “ATMPs”, and “clinical trials”, each bounded by its own legal definition and bespoke sets of regulatory rules of production, storage, use, and market approval.’^14^ And so it is with medical devices at both UK and EU level. The relevant Regulations and Directives are constructed in such a way that the objects (the devices themselves) rather than subjects (persons into whom they are implanted or to whom they are attached) are the primary focus of regulatory attention.^15^ Specifically, the law takes a ‘bounded object’ approach,^16^ constructing medical devices as different types of objects: ‘risk objects’, ‘marketised objects’, ‘innovation objects’, and so on.^17^ These constructions will be unpacked later in this section. For example, we will see that the framing of medical devices within the relevant regulatory instruments is intimately connected the functioning of the EU internal market. This situates medical devices firmly in a marketised context. We argue that the consequence of this and other regulatory framings is that, to the extent that persons factor into the regulation and governance regarding medical devices, they do so in relation to these particular object-focused frames of reference. This, as we will also see, is problematic.

#### 1. Regulatory Context

Currently in the UK, medical devices are governed by the Medical Devices Regulations 2002. These implement and give effect to three European Directives: (i) Directive 90/385/EEC concerning active implantable medical devices (AIMDD), (ii) Directive 93/42/EEC concerning medical devices (MDD), and (iii) Directive 98/79/EC on *in vitro* diagnostic medical devices (IVDD). In April 2017, after a complex period of drafting, negotiation, and amendments, the EU published two new Regulations, which repeal and replace the three previous Directives. These are Regulation (EU) 2017/745 (Medical Device Regulations—MDR) and Regulation (EU) 2017/746 (*In Vitro* Diagnostic Regulations—IVDR). Both of these came into force in May 2017 and will be fully implemented by May 2020 and 2022, respectively. However, their influence on the UK largely remains to be seen given uncertainties around the exact shape of Brexit. At the time of writing, the provisions of the European Union (Withdrawal) Bill 2017 would mean that ‘EU-derived legislation’ that has been separately written into UK law will have the same force it had the day before ‘exit day’.^18^ As such, the three medical device Directives will continue to have force in the form they are found in the UK’s Medical Device Regulations 2002. Whether or not any of the provisions of the new MDR and IVDR will have direct influence on future UK law mainly depends on whether they are explicitly written into law prior to exit day.^19^ This is because most of these are due to come into force after exit day. This will either be the date of a withdrawal agreement or 2 years after Article 50 was triggered (thus 30 March 2019).^20^

An overhaul of the EU’s approach, and the resultant Regulations, was deemed to be necessary because of apparent lacunae in the Directives, including the need for better pre- and post-market scrutiny.^21^ Some of the changes in particular can be seen as a response to safety and product liability concerns arising from device failures such as defective DePuy metal-on-metal hips and Poly Implant Prothèse (PIP) breast implants. The former involved the higher than normal revision rates for DePuy Articular Surface Replacements and total metal-on-metal hips, something which has been described as ‘one of the biggest disasters in orthopaedic history’.^22^ In 2012, a Medicines and Health Regulatory Agency (MHRA) device alert encompassing all metal-on-metal hips (not just DePuy’s) was issued,^23^ and in 2013 the NHS stopped using metal-on-metal hip joints.^24^ In the same year, the DePuy alerts were issued, the MHRA published a device alert in relation to silicon breast implants manufactured by the French company PIP. French regulatory authorities discovered that the PIP had been using a non-approved silicone gel for the implants^25^ and subsequent investigations revealed that these implants had a significantly higher likelihood of rupture (by two to six times) than other breast implants.^26^ The high-profile nature of these medical device scandals helped to drive calls for change at EU level.^27^

We do not want to downplay the significance of robust product safety and risk regulation in relation to medical devices. This is of the utmost importance, as both the DePuy Hip and PIP breast implants scandals illustrate. Nevertheless, a very particular approach to medical devices, which constructs them as different types of objects, is at play in EU and, consequently, the UK legal and regulatory contexts. This is something we have reason to be cautious about when it comes to everyday cyborgs. In what follows, we suggest that in being overly object-focused (which we will see through the different constructions of medical devices as regulatory objects), the law is insufficiently attentive to the conjoined future for which such devices as destined; that is, the implications of the assemblage of subjects and objects which everyday cyborgs represent.

#### 2. Constructing Medical Devices as Law’s ‘Objects’

A principal way in which the relevant regulation and legislative instruments construct medical devices is as ‘risk objects’. As defined by Hilgartner, ‘risk objects’ are ‘things that pose hazards, the sources of danger, the entities to which harmful consequences are conceptually attached’.^28^ The focus on risk and safety is explicit in the medical device Directives. Consider, for instance, Annex I of the MDD:

[D]evices must be designed and manufactured in such a way that, when used under the conditions and for the purposes intended, they will not compromise the clinical condition or the safety of patients, or the safety and health of users or, where applicable, other persons, provided that any risks which may be associated with their intended use constitute acceptable risks when weighed against the benefits to the patient and are compatible with a high level of protection of health and safety.^29^

Similar wording can be found in the AIMDD.^30^ The 2002 Regulations in the UK were created under powers deriving from the Consumer Protection Act 1987 and, given this, safety provisions within the Regulations, are made in reference to the Act.^31^ In this respect, ‘safe’ is defined as ‘no risk, or no risk apart from one reduced to a minimum, that [the goods] will cause the death or, or any personal injury to, any person whatsoever’.^32^

The new EU Regulations continue, and indeed strengthen, this focus on risk and safety. This can be seen both in the Recitals and the Articles.^33^ Recitals 1 and 2 of the MDR, for instance, state that ‘a fundamental revision of those Directives is needed to establish a robust, transparent, predictable and sustainable regulatory framework for medical devices which ensures a high level of safety and health whilst supporting innovation’ and ‘this Regulation sets high standards of quality and safety for medical devices in order to meet common safety concerns as regards such products’. Almost the exact same wording is found in the IVDR.^34^ The Regulations also provide for new classification systems for devices (which are explicitly risk-based),^35^ the requirement to supply more robust clinical data and evaluations of devices,^36^ and increased requirements for post-market surveillance.^37^ As noted by Flear in respect of the regulation of new health technologies in general, the EU’s regulatory techniques

… narrow the meaning and framing of technological risk to being principally about product safety at different stages of product development and ultimately marketing within the internal market. At the same time the techniques bracket off and marginalize the other kinds of harms or hazards to which risk might pertain.^38^

In this manner, the way in which risk is constructed, and the focus on risk objects is related to the framing of medical devices as ‘marketised objects’.

This framing stems directly from the economic motivations which permeate the EU’s (and hence the UK’s) regulatory approach, as well as its specific competences in relation to health. In short, there is a strong connection between the development and framing of the relevant EU law and the goal of supporting and growing the EU internal market.^39^ Moreover, health is not an exclusive EU competence. Indeed, the only specific Treaty base for health provides for supporting or coordinating action, and for a high level of health protection to be taken into account under its other powers; for example, the internal market or consumer protection.^40^ Accordingly, health and innovation (in healthcare technologies) are not framed solely as goods in themselves; there is also a marketised dimension to them. Thus, a number of key EU strategy documents contain explicit economic rationales for the pursuit of both health and innovation within the health sector. Consider, for instance, the tagline of the Innovation Union (a Europe 2020 initiative): ‘Innovation Union is the European Union strategy to create an innovation-friendly environment that makes it easier for great ideas to be turned into products and services that will bring our economy growth and jobs.’^41^ Note how an economic rationale runs through all four reasons for including a health policy focus in Europe 2020:

- keeping people healthy and active for longer has a positive impact on *productivity and competitiveness;*
- innovation can help *make the healthcare sector more sustainable* and find new cures for health conditions;
- the healthcare sector has an important role to play in *improving skills and creating jobs* as it employs 1 in 10 of the most qualified workers in the EU;
- with a projected 45% increase in the number of people aged 65 and over in the next 20 years, *financing rising healthcare costs* and access to a dignified and independent life for the ageing population will be central to the political debate.^42^

The three EU Directives, the 2002 UK Regulations, and the new EU Regulations only serve to reinforce this view of the regulatory approach. These are not framed in terms of health promotion or improving health by the use of such devices. Instead, the focus is on the conditions for the bringing to market of medical devices and health protection via safety and risk management procedures.^43^ Within such a framework, competitiveness and market advantage rather than improving health (or other social goals) *per se* become the drivers of innovation.^44^

And here we find yet another dimension to the framing of medical devices. They are often constructed as ‘innovation objects’. As noted by Stokes:

Law and regulation are associated with control and the imposition of constraints on behaviour, whereas science and technology are imagined as endless sources of creativity and innovation. Although these are gross caricatures, they still animate many of the policy debates on the regulation of new technology—particularly debates that centre on the potential heavy-handedness of regulatory activity.^45^

In relation to medical devices, the EU emphasises the role of innovation in the sector and the importance of the ‘innovativeness’ of healthcare and healthcare solutions.^46^ Such framing is, as Stokes has noted of the Innovation Union in general, part of the ‘EU’s political identity’.^47^ This comes through in the new Regulations, albeit intimately connected to concerns regarding both safety and the functioning of the internal market. Both the MDR and the IVDR refer to ‘a high level of safety and health whilst supporting innovation’ and ‘the smooth functioning of the internal market as regards medical devices’.^48^ It may be that the kinds of devices under consideration here, at least in their embodied forms, are far from the minds of EU law and policymakers when they discuss innovation.^49^ This, as we are about to see, is part of the trouble with the different framings of such devices in law and regulation.

Undoubtedly, there are other ways beyond their constructions as risk, marketised, and innovation objects in which law and regulation frames medical devices. Our intention was to give a flavour of these different constructions in order to point to a greater risk (pun intended); that is, that there can be a neglect of important considerations which are not object-focused. In particular, the subjects—the end users of medical devices—can become marginalised or overlooked.^50^ One such example is consent processes for the use and implantation of medical devices. Arguably, consent processes in healthcare in general are impoverished. The consent transaction as currently practiced is binary in nature.^51^ A person either gives or withholds consent at the time a procedure (e.g. implantation of a pacemaker) is due to take place (or in advance of this, reaffirming it nearer the time). This, however, misses crucial aspects of what Laurie terms ‘consent as *process’*;^52^ specifically, it neglects the ‘temporal and spatial dimensions’ of the subject’s experience.^53^ In the context under discussion here, this means persons experiences of *becoming* and *being* everyday cyborgs.

By constructing medical devices as risk objects, for instance, we fix them in time and substance. They are what they are at the time of implantation: objects discrete from persons. They are conceptually removed from the embodied (or at least conjoined) future for which they are destined. As such, they are disconnected from, and in essence rule out, potential legal futures; that is, by framing and fixing in a particular way, alternative legal approaches, which are less object- and more subject-focused, are excluded.

Relatedly, there is also a sense in which the risks associated with medical devices, or at least the available regulatory responses to these, are also fixed, or at the very least much stagnated. We say stagnated because they are fixed within the operative regulatory landscape until such a time as they are revised either in response to a large-scale scandal (such as the DePuy hip failures) or until incremental changes are made through systems of post-market surveillance, something which can be slow-moving. Yet in reality risks relating to attached and implanted medical devices are ever-changing; something which the increasing ‘smartness’ of certain devices may bring to the fore. Consider, for instance, an integrated device with wifi. Such a device may be able not just to process and send data to servers, but to receive updates—software and informational. This two-way traffic represents a device in a state of flux (not to mention there may also be feedback loops internal to the devices functioning). Plausibly the risks associated with such devices are also subject to constant change, potentially in response to the day-to-day activities or physiological state of the everyday cyborg.^54^ Whilst new post-market surveillance procedures mandated by the MDR and IVDR might help to address regulatory stagnation to a certain degree, it, nevertheless, does not provide an altogether satisfactory framework (either conceptually or practically) for thinking about medical devices or everyday cyborgs.

Conceptually speaking, the object focus is problematic, because, as Hilgartner argues:

[T]he world does not present itself prepackaged into unambiguous and clearly-differentiated objects. On the contrary, the division of the world into objects is a conceptual achievement. The world can be parsed in many different ways, and category systems used to classify objects are fundamentally ambiguous.^55^

Moreover, as Section II demonstrated, and we will see further in Section IV, the transgression of the bodily boundary and the re-formation of persons as *integrated persons* means that neither the object nor the subject ought to be construed as fixed. As Hoeyer observes, ‘technologies using and engaging with “the human body” challenge ideas about body boundaries … [these] are not given and never have been – they are under constant establishment, biologically as well as culturally.’^56^ Becoming and being an everyday cyborg involves the deconstruction and (re)establishment of boundaries: it involves process and transformation, something not captured by the current regulatory approach.^57^ These processual aspects are important if we think that the (approach of the) law ought to be reflective of the reality of people’s lives. Laurie has highlighted the importance of this in his discussion of health research regulation. He says:

The attention of law and regulation on ‘bounded objects’ such as personal data and tissue should be questioned on at least two counts: first, for the fallacy of attempting to ‘fix’ such regulatory objects, and to divorce them from their source and the potential impact on identity for the subjects themselves; and, second, for the failure to see such objects as also experiencing liminality [being in between states and as undergoing transformation]. Both of these points suggest a need to approach the management of regulation in processual and potentially transformative terms.^58^

Practically speaking, in constructing and fixing medical devices as different types of ‘objects’, current law and regulation may simply not be flexible or responsive enough to deal with changing and unforeseen risks arising from the joining of persons with integrated devices.

### B. Damage to Devices and Liability

The difficulties for the law regarding everyday cyborgs are also evident when we think about the issue of damage to devices. How ought the law to deal with damage to a highly integrated medical device or prosthesis? Ought it to view this as personal injury, damage to property, or something else? Medical devices and prostheses begin life unambiguously as external things. As such, they are uncontroversially viewed as items of personal property (chattels).^59^ They are transferred, bought, and sold, and those who possess them are protected by the usual operation of personal property law. The underlying conceptual question to be answered, therefore, is whether these devices retain their property status upon implantation or attachment, somehow lose it by becoming part of the living body, or acquire some sort of hybrid status. The answers to such questions are not straightforward.

An old Health Notice issued in 1983 by the then Department of Health and Social Security (DHSS) states that

On implantation, an implant becomes the property of the person in whom it has been implanted and it remains his or her property even if it is subsequently removed. Following the patient’s death, it forms part of his or her estate unless there is any specific provision to the contrary.^60^

This is a position that has more recently been re-stated by the MHRA^61^ and can also be found in advice from various medical negligence firms.^62^ Despite its acceptance by these organisations, the policy is of questionable legal provenance and usefulness. Not only does it have no firm basis in any established law regarding either persons (living or deceased) or medical devices (or things more generally), it almost certainly runs counter to it.

As noted by Dickenson, the law considers that something is either a person (subject) or a thing (object), ‘but not both’.^63^ Although it is unusual to find explicit statements within the law to this effect, it can be clearly seen in its internal structure. Broadly speaking, the law is divided into that which relates to persons (e.g. assault and battery, personal injury, medical negligence, etc.) and that which relates to things in the external world (e.g. land law, personal property, sale of goods, etc.).^64^ There is thus an implicit, yet foundational, incorporation into the law of a subject–object dichotomy. The specific question of the property status of medical devices once implanted into or integrated with the body has not been tested in the courts. Nevertheless, in *R v Bentham* we do find a statement, albeit in a different context, which is reflective of this general position. This question at issue, as put by Lord Bingham, was whether

a person who has his hand inside a zipped-up jacket, forcing the material out so as to give the impression that he has a gun, [could] be held to have in his possession an imitation firearm within the meaning of section 17(2) of the Firearms Act 1968.^65^

The answer for the House of Lords in this case was no, in particular, that a person’s fingers could not be considered to be property.^66^ Part of the reasoning was as follows:

One cannot possess something which is not separate and distinct from oneself. An unsevered hand or finger is part of oneself. Therefore, one cannot possess it … What is possessed must under definition be a thing. A person’s hand or fingers are not a thing.^67^

Hence, the law regarding property entails a normative prerequisite of separation from persons,^68^ that is, a division between subject and object. Pragmatically, this may not be completely insurmountable for the law, although we will suggest shortly that it is conceptually problematic.

In the context of prostheses, Goold and colleagues argue that both personal injury law and property law could be applicable depending on the device and circumstances at hand. Conceivably, the most straightforward situation relates to an unattached prosthesis. In terms of the criminal law, the Theft Act 1968 would apply if stolen and the Criminal Damage Act 1971 and the Criminal Justice Act 1983 if damaged.^69^ Meanwhile, civil liability for damage would lie with the torts which protect property (conversion, trespass to goods, and negligence).^70^ Where attached to and integrated with the living body, Goold and colleagues argue that damage done could be considered as an offence against the person. They point out that common assault does not require actual bodily injury, and so damage or an attempt to damage an attached prosthesis could be classified thus.^71^ On the civil side, trespass to the person and negligence could both be available routes for redress.^72^

The main difficulty is establishing whether appropriate redress (including penalties and compensation) is available via the different legal routes.^73^ There is a marked difference in the law’s approach to damage to property and harm to persons. The criminal law treats harm to persons much more seriously than damage to property. Whilst the penalties available for criminal damage to property and offences against the persons (such as assault) both depend on the degree of damage/harm (as well as aggravating factors), the scope and scale of penalties (as we move from minor to more serious crimes) are *generally* greater in the case of offences against the person. At the very lowest end, the penalties are arguably comparable. Compare, for example, criminal damage to property not occasioning actual damage (or where the damage is not lasting/serious) with common assault and where no actual bodily harm is inflicted (or where there is no lasting/serious harm). For the lowest level of criminal damage, the maximum penalty is a Band C fine (125–175% of an offender’s weekly income).^74^ In the case of the lowest level of common assault, the maximum penalty is the same.^75^ As the seriousness of the offences increase, however, the difference between damage to property and harm to persons becomes apparent. Consider moderate and significant criminal damage to property. If moderate damage to property, the maximum penalty is a medium-level community order. If significant damage, it is 12 weeks custody.^76^ Compare this to the two lowest categories of assault occasioning actual bodily harm. The maximum penalty for the least serious offences is a high-level community order. The next level (entailing greater harm and lower culpability or lesser harm and greater culpability) can attract a custodial sentence of up to 51 weeks.^77^

Likewise, with regard to tort law, although there is a sliding scale regarding recompense, depending on the exact details of individual cases, in general, harm to persons attracts higher levels of compensation than damage to property. Damages for torts relating to property and those relating to persons aim to put the claimant in the position they would have been had the wrong not been committed.^78^ Negligent damage to goods is compensated either by the cost of repair^79^ or that of the replacement of the goods.^80^ A variety of consequential losses may also be compensated. In the case, for example, of a damaged car, the claimant may be compensated for the repair or replacement of the car, as well as losses such as those incurred in hiring a car^81^ or loss of profits consequent on not being able to use the car.^82^ With regard to personal injury both pecuniary and non-pecuniary losses may be compensated. The range and scope of the damages which may be awarded are, however, much broader. And, consequently, the overall level of damages awarded potentially much higher. Damages for pecuniary losses can include loss of earnings,^83^ medical expenses,^84^ buying special equipment or moving to special accommodation amongst other things.^85^ Damages may also compensate for non-pecuniary losses, usually (i) loss of amenity and (ii) pain and suffering. These are awarded on a tariff basis, for instance, from £1,950 for certain orthopaedic injuries to £322,060 for very severe brain injuries.^86^

The relevance of all this for the everyday cyborg is that viewing a prosthesis (or indeed other medical devices, implanted or otherwise), as objects of property may not offer adequate redress for damage done. The reason being that remedies available for either criminal or negligent damage to property may not capture the vital functions (mechanical, physiological, or otherwise) that these devices serve for persons. Moreover, they do not take account of the incorporation into (the lives of) persons which they represent (more on this in Section IV). In the case of a one of a kind sophisticated neuro-prosthesis, for instance, even the replacement value of the device plus some consequential losses may not appropriately capture the loss to the person of the original and thus the wrong(s) done.

The challenge is captured well by Laurie’s description of the ‘impositional nature of law’.^87^ Law, ‘by its nature, seeks to establish structures, fix regulatory objects, assign responsibilities, and attribute liabilities’.^88^ This impositional nature is highlighted by the discussion in this section. When asking how the law would deal with challenges related to damage, it becomes clear that we are required to ‘fix’ the thing in question as either a subject or an object in order to assess which part of the law is most apt to deal with it. In one sense, this is simply pragmatic. The law needs to find a resolution to a variety of conflicts and to work within its existing structure to do so. This will be illustrated further in Section III(D) when we discuss biohacking. There we will see that, although a wide array of either subject or object-based liabilities may be available for harm ensuing from hacking, they would likely still follow the usual well-delineated subject or object-focused pathways. Although pragmatic within the current constraints of the law, it is not quite satisfactory when advancing technology challenges the very ontological nature of such divisions, as is the case with integrated persons. In essence, challenges such as these fall outwith the operative boundaries of the law’s current conceptual framework. This is not to say that the law cannot deal with such issues if and when they arise. The ability to do so and to adapt accordingly over time is one of the strengths of a common law system, and as Goold and colleagues’ argue, the law as it stands could be made to work in this area. Nevertheless, as we will demonstrate in Section IV, a re-analysis of the law, along with the conceptual and philosophical terrain underpinning it, may still be needed.

### C. Data and Privacy

Integral to the everyday cyborg are integrated goods. As explained by Thomas, these are ‘goods which have a high level of integration between the physical functionality of the goods, and the software enabling the functionality’.^89^ For example, many medical devices such as pacemakers and implantable cardioverter defibrillators now have integrated software which enables the device to diagnose when a condition requires therapy. Further, they can control the delivery of therapy to the patient’s body. This integration of hardware (tangible) and software (intangible) is crucial. Albeit in a different context, as noted by Scott Baker J in *St Albans City & District Council v International Computers Ltd*, ‘[b]y itself hardware can do nothing. The really important part of the system is the software. Programs are the instructions or commands that tell the hardware what to do’.^90^ Amongst other things, the functionality added by software and wifi capabilities enables devices to collect, store, and transmit data about patients’ health, as well as data about the status and function of devices themselves.

This data collection can be done in a number of different ways. For instance, a patient’s healthcare team can extract information during an outpatient hospital visit. Alternatively, where the device is equipped with remote monitoring technology, the data can be transmitted automatically via a wireless connection to a secure website; subsequently, this data can then be accessed by the healthcare team (patients can also manually start data transfer if they are experiencing ‘symptomatic episodes’).^91^ This allows the patient’s condition and device to be monitored remotely by healthcare professionals. The data collected by the device could include identifying information about the patient such as their name and date of birth, information about the their health status such as vital signs, diagnosed conditions, and therapies, and data relating to the device’s function, for example, battery status, lead impedance, disabling of therapy, and inadequate safety margins for sensing or capture.^92^

Where prostheses and implants collect, store, and transmit data, questions relating to the control of data and privacy come to the fore. Although data and privacy concerns regarding other types of healthcare data, such as electronic records, have received a reasonable amount of attention,^93^ the everyday cyborg context has not. To help to think about what the difficulties here might be, consider the story of Hugo Campo from the USA. Hugo suffers from heart disease and has an ICD.^94^ This collects data about its own function and Hugo’s health status, and the data is transmitted to the manufacturer of the device. Medtronic, the manufacturer, then processes the data and sends the results to the Hugo’s doctor through a web portal. The information is not sent to the patient himself. Whilst patients who want access to the data from their ICDs can pay for biannual consultations with their doctors to obtain short summaries of the data, Hugo and a number of other heart patients want direct and immediate access to their data. Requests to this effect have, however, been denied by Medtronic.

Medtronic say that under the Food and Drugs Administration (FDA) regulations, they are permitted to send reports to doctors through their web portal, but not to patients.^95^ They maintain that they will require the FDA approval to send the data to patients and, even with such approval, there are other regulations that might restrict them from delivering the data.^96^ They also raise the issue of costs, claiming that they will need to create a website specifically for patients, which will be costly, particularly because according to them, there are not an overwhelming number of patients who want direct access to their ICD data. Additionally, there also appears to be a concern that patients might misunderstand and misinterpret the data.^97^ Perhaps unsurprisingly, some patients disagree, contending that the data from ICDs should belong to them because it is their bodies, hearts, and data. They also say that regular feedback will enable them to become better informed about their health, helping them to manage their health more effectively.^98^

Whilst this particular story is from the USA, it highlights tensions regarding the ownership and control of data generated and collected by medical devices. In the UK, the question of who ought to have control over data collected from prostheses and implants does not necessarily have an easy answer. This is because the sheer range of data collected by such devices means that the existing legal framework may not give adequate cover or protection to the everyday cyborg in respect of data usage. For brevity (and because the law relating to data protection is now voluminous and complex), we restrict our comments to three issues: (i) data access; (ii) exemptions and derogations in relation to research usages; and (iii) the significance of different data types.

The Data Protection Act 1998 (DPA), for now, is the main source of data protection law in the UK. This implements the 1995 EU Data Protection Directive (Directive 95/46/EC). However, a new General Data Protection Regulation (GDPR) (Regulation (EU) 2016/679), which will replace the Directive, was adopted in April 2016 and comes into force from May 2018.^99^ Like the MDR and the IVDR, those parts of the GDPR in force before exit day would automatically become part of UK law in virtue of the provisions of the European Union (Withdrawal) Bill. Nevertheless, the UK has already drafted a Data Protection Bill (DPB) that aims to implement the GDPR. This began its passage through Parliament in September 2017 and deals with the GDPR, as well as supplementary issues not covered by it. This is necessary, because, regardless of Brexit, compliance with the standards set out in the GDPR will be required if data flow is to be permitted between the EU and the UK.

For the purposes of the DPA 1998, ‘personal data’ are data from living persons who can be identified either from that data or from a combination of that data and other information already held.^100^ Data about a person’s physical or mental health are deemed to be ‘sensitive personal data’.^101^ Similarly, under the DPB, personal information ‘means any information relating to an identified or identifiable living individual’.^102^ The GDPR includes ‘online identifiers’ such as IP addresses and cookies as part of personal data, in effect expanding what counts as such.^103^ Whereas the 1998 Act has a special category of ‘sensitive data’, which includes health data, the GDPR and DPB refer to ‘sensitive processing’, thus shifting the emphasis from the data to what happens to it.^104^ The processing of biometric, health, and genetic data is included in this.^105^

Under the GDPR and the DPB, like the 1998 Act, individuals have a putative degree of control over the use of their data. The GDPR is clear that consent is needed for the processing of personal data.^106^ This must be explicit with regard to special categories of data, including health data.^107^ The 1998 Act gave individuals a right to access regarding any personal data held about them, this carries on in the GDPR and DPB.^108^ Additionally, the GDPR creates a new right of portability, meaning that persons have the right to have their data transferred from one data controller to another ‘in a structured, commonly used, machine-readable and interoperable format’.^109^ Although formulated to enable transfer between different IT environments (e.g. cloud-based storage systems), there is no reason in principle why this right would not apply to device-mediated everyday cyborg data. Hence, in the UK, everyday cyborgs such as Hugo could apply to see any health-related data held about them which has been downloaded from their devices (be this by a hospital or manufacturer or some other organisation).

Despite these provisions, arguably the 1998 Act and the GDPR take a research friendly approach and, in so doing, allow exemptions and derogations that potentially weaken the putative control of individuals regarding their data. Both permit health data to be processed, subject to certain safeguards,^110^ without the need for explicit consent if the processing is necessary for medical purposes, including for research purposes.^111^ Article 89 of the GDPR permits potentially broad derogations in this respect. As such, the EU or Member States could derogate in respect of rights of access, rectification, restrictions on processing, and objections to processing (as long as measures to respect the principle of data minimisation—such as pseudonymisation—have been put in place; that is, that processing is ‘adequate, relevant and limited to what is necessary in relation to the purposes for which they are processed’).^112^ Consequently, although the GDPR may well meet one of its principal aims, which is to give individuals greater control over their personal data, it remains to be seen whether it goes far enough.^113^

One aspect of particular interest is the question of the types of data generated and collected specifically by medical devices. It is debatable, for instance, whether data collected about device status and function can be classified as personal, or its processing deemed as sensitive. At first glance, it might seem like data about device functioning would not be covered by the 1998 Act, the GDPR, or the DPB and would fall outside the scope of the everyday cyborg’s limited control. Although it comes from devices implanted into or attached to persons, it is not information that relates to the living individual (the subject), but rather it relates directly to an object (the medical device or prosthesis). However, the conjoined subject–object context which everyday cyborgs represent challenges the subject–object divide represented by the division of data types (personal versus device data). There are at least two reasons for this.

First, information regarding device functioning is undoubtedly of use to manufacturers (as an aid to identifying problems with devices) and unknown future patients might benefit from the development of improved devices. However, it is also plausible not only that current patients have an interest in what such data can tell them, but that it might be directly useful to them. Patients might have an interest in better understanding how their bodies and health condition interact with or affect device functioning; for instance, whether devices perform better or worse (or just the same) in situations where they are under increased physiological stress.^114^ If this is correct, then it at least raises the possibility that device data ought to be treated as ‘personal’.

Secondly, there is an ontological and conceptual dimension to maintaining a distinction between data types in the conjoined context. In viewing implanted medical devices and integrated prostheses solely as objects somehow separate from persons (despite physical and functional integration), we set them apart from those persons. Yet the assemblage of integrated persons and integrated devices signifies an ontological blurring of boundaries between subject and object, which the conceptual foundations and structure of the law have not hitherto had to account for. For everyday cyborgs, it is simply not obvious that data about how medical devices function ought to be treated all that differently from physiological data about how the body is functioning. Part of the intuition that there is a difference here may have as much to do with perceived distinctions between the biological and the synthetic as it does with those between subject and object. We will return to this aspect in Section IV.

###  D. Security and Biohacking

In recent years there has been a growing movement of people who have been ‘hacking’ their bodies (so-called biohacking); that is, altering or augmenting their bodies through a number of DIY methods, including by the use of implanted and wearable devices. Usually this is done without the involvement of a medical professional, and outside the healthcare setting. The aims of those using technology in this way are diverse. Some do it to improve their health, others to enhance their senses or extend their human abilities. Included in this are those who have modified their medical equipment to add new functions.

One example of a biohacker is Neil Harbisson, an artist and cyborg activist, who has an antenna implanted in his skull that allows him to *hear* colour. Neil was born with achromatopsia, and as a result of this condition, he can only see in shades of grey. The antenna’s internet connection enables Neil to receive colours directly into his head through external devices such as mobile phones or satellites.^115^ Another example is the #WeAreNotWaiting movement. Here people with diabetes are exploring various methods of using technology to improve the management of their condition. Some of these have developed their own homemade ‘artificial pancreas’.^116^ For instance, Dana Lewis and her husband developed an artificial pancreas to help manage her Type 1 Diabetes. They did so because Dana was unhappy with the limitations of medical devices on offer. She did not think they functioned adequately in measuring and controlling her blood sugar levels.^117^ The couple hacked into Dana’s glucose monitor, modified it, and connected it to her insulin pump. They created a closed loop system that can predict what her blood sugar level will be some hours into the future and then automatically deliver the correct dose of insulin. They have made the source code available online, and device manufacturers are currently developing new devices using their prototypes.^118^

A potentially more troubling instance of biohacking would involve the malicious hacking of a person’s medical device by third parties. As implanted medical devices have become more sophisticated, concerns have grown regarding potential security vulnerabilities that would allow them to be hacked. In 2012, a US Government of Accountability Office report identified the potential for security threats from a number of medical device vulnerabilities, including unauthorised access, malware, and denial-of-service attacks.^119^ Moreover, security specialists and hackers have demonstrated that the remote hacking of devices such as pacemakers is not only possible, but relatively easy.^120^ Concerns about hacking, especially the possibility of being assassinated via a malicious attack, prompted former US Vice-President Dick Cheney to have the wireless capabilities of his pacemaker disabled.^121^

Both types of biohacking draw attention to potential difficulties for the law. The first, which we will outline in the next section, is whether the actions of DIY biohackers who alter medical devices infringe intellectual property rights held in respect of such devices and their software. The second relates to liability for harms that might flow from either the direct modification of devices or from making available the source code to allow others to modify devices. The third centres on malicious interference with either devices or wireless communications to devices.

Where modifications to devices and prostheses are done by those who are not healthcare professionals (or the manufacturers), they are in essence operating outwith the limits and protections of both medical device regulation and healthcare law. As such, biohackers and the products they develop circumvent or contravene law and regulation that has been developed with product safety in mind.^122^ A principal concern in such scenarios is that of liability for harms which flow from modified devices. Consider this in terms of the functionality of relevant devices. Many technologies of the everyday cyborg contain software that enables the devices to monitor and diagnose particular conditions. They can also control the subsequent delivery of a therapy, for example, an electric shock from an ICD or insulin from an insulin pump. In such instances, a malfunction could pose significant risk to the patient, perhaps causing a failure to detect when therapy is required or delivering inappropriate therapy (or indeed none at all). Such malfunctions could result in serious injury or even death in certain circumstances.^123^ Think, for instance, of an ICD that fails to detect an irregular heart rhythm, or worse, fails to deliver a life-saving shock to a patient’s heart when it goes into a life-threatening rhythm. Consider also a myoelectric prosthetic arm that malfunctions when the user is driving, thereby causing an accident and endangering other road users as well as themselves.^124^

Ordinarily medical devices and other relevant product liability law would be applicable in those instances where there is a design or manufacturing defect in a device.^125^ However, it is not clear what the situation is where a biohacker (either the everyday cyborg themselves or a third party) alters a product after it has left the manufacturer.^126^ If a person suffers harm from using the modified product, who ought to be liable? Would a finding of liability against a DIY biohacker be mitigated by the existence of the security vulnerability that enabled them to alter the device? Would this make the manufacturer partially liable? Furthermore, where the source code has been released (as in the case of the hacked glucose monitor) and other people use this code, who would be liable for any resultant harms?

In the case of malicious interference and third-party hacking, at least initial recourse might be found in the Computer Misuse Act 1990. The Act contains a number of offences which could be applicable in the case of medical devices, including that of unauthorised access to computer material, acts which impair the operation of a computer, and acts causing (or risking) serious damage.^127^ Quite deliberately the Act, and subsequent amendments to it, do not specify what a computer is, and it seems likely that the types of medical devices discussed here would fall within the Act’s ambit. Nevertheless, the use of this Act might not adequately account for the consequences of hacking in the everyday cyborg situation. Because the Act is object-focused (i.e. computers), its sanctions, in the form of prison terms and fines, are in line with this^128^ and not as serious as when harm to persons occurs.

In the everyday cyborg context, hacking devices *is* to a certain extent hacking persons, and the consequences of this could be serious. Think, for instance, of an ICD that fails to detect an irregular heart rhythm, or worse, fails to deliver a life-saving shock to a patient’s heart when it goes into a life-threatening rhythm. As such, we need to ask what other routes would be available for harms done by hackers. In principle at least, the whole array of both object-focused liabilities—such as those stemming from the Computer Misuse Act—and subject-focused ones^129^—such as assault or even involuntary manslaughter or murder^130^—ought to be available for harm done. Murder, at first glance, appears reasonably straightforward. So long as the requisite intent to do harm is present, and causation can be proven, there are no bounds on the *mode* of killing for the offence to obtain. Yet, proving causation might be tricky depending on both the circumstances surrounding death and how easy it is to tie the hacking to that death. Lesser offences such as involuntary manslaughter might be even more difficult. Take, for instance, a scenario where a pacemaker is hacked with the intent to improve its functioning, but instead the person dies. Or consider cases where persons who utilise the altered source code for their insulin pump die. Here there is no malicious intent to cause serious harm so a lesser offence than murder would need to be pursued. A successful prosecution for involuntary manslaughter would have to demonstrate both causation and that the hacker undertook an objectively dangerous act. The same problems with causation are present here as with murder. But there is also the matter of proving that the hacking was objectively dangerous. Given the novelty of the question that would be facing the jury in such cases, they would have to be persuaded that a reasonable person would have thought the course to action at issue to be dangerous. It is not a given, however, that they would be so persuaded.

### E. Intellectual Property Rights

The prostheses and implants attached to the everyday cyborg incorporate two types of property rights: those attaching to (i) the tangible object (property in chattels) and (ii) intangible aspects such as the branding or design of the device, or the software incorporated in it (intellectual property—principally patents, copyright, design rights, and trademarks).^131^ The law treats these as separate interests, and the regimes governing them are essentially separate. Ordinarily, property rights in chattels protect owners in their use and control of the physical objects in question, whilst intellectual property rights (IPRs) pertain to the owners’ interests in the trade secret, invention, or tangible expression that is embodied in a product.^132^ In the context of the everyday cyborg, the hardware components of an implanted medical device or attached prosthesis might be protected by a series of patents, design rights, and trademarks. And where devices are capable of running software, this might be subject to copyright.^133^ What we will see in this section is that the nature of intellectual property as rights over intangibles, coupled with the possibility that various IPRs could be attached to goods and/or embedded aspects of goods, gives rise to several potential difficulties.

One important question is who owns, or ought to own, and control the IP in these devices once they become attached to the person? Do the IPRs remain with their originators, or do they pass to the persons into whom they are implanted or to whom they are attached? Establishing clarity (which we suggest we do not currently have) on IPRs in medical devices and prostheses is important for a number of reasons: for instance, to ascertain the potential downstream control that an IP holder will have as the device goes through a series of transfers (ordinarily set out in end-user licence agreements), whether these are to healthcare organisations purchasing the devices or patients themselves once implanted or attached. It might also be required in order to ascertain rights to access for the purposes of software upgrades or repair.^134^ Alternatively, there may be occasions where it is necessary to identify restrictions (which flow from the IPRs) on what users can do with the devices, particularly with regard to users modifying software or installing software that is not approved by the manufacturer of the device. In such scenarios, clarity is also needed on what the manufacturer is permitted to do where such modifications are made. As we saw in the previous section, this is especially important in the case of medical devices because of the potential harm that could be suffered by a user if a device malfunctions.

Since the law treats the IP and property rights in the goods in which they are embodied as separate interests, the sale of these goods does not usually involve transfer of the IPRs to the purchaser.^135^ An owner can transfer use and control of IP in a number of ways, each of which entails different proprietary consequences for the original holder of the IPRs and those who subsequently come into possession of them. The detail and requirements (for the legal validity) of the transfers differs depending on the types of IPRs (i.e. whether they are patents or copyright and so on).^136^ Broadly, however, IP and rights contained therein can be transferred by assignment (i.e. voluntary transfer including sale and gratuitous transfers) or by bequest. They may also be licensed or mortgaged.^137^ Since assignments of IPRs are distinct legal transactions that entail certain formalities in order to be valid, and these are not met by the *mere* transfer of physical devices,^138^ purchasers and other users (such as everyday cyborgs) are restricted in their usage of such devices. Even with a valid licence, restrictions apply. Usually licensees can use the subject of the IPRs pursuant to a set of terms and conditions; for example, on the condition that they pay a set fee and a percentage of royalties to the licensor (the IPR holder). Licensing does not involve the transfer of ownership. The IPR holder retains ownership of the intellectual property.^139^ In the case of the everyday cyborg, this becomes problematic on a number of fronts.

The question of the transfer of IPRs takes on a new significance when it comes to this assemblage of integrated persons and integrated goods. To wit, the transfer of a prosthesis or implant, which embodies components that are subject to patent, design, and/or copyright law, does not (on the usual operation of IP law) involve the transfer of the IPRs to the purchaser of the device (hospitals, etc) or indeed to the everyday cyborg once implanted/attached. Given this, parties acquiring such devices, including everyday cyborgs themselves, will be bound by the restrictions of the IPRs and any licensing terms and conditions. In terms of embedded software, the use of such software is often subject to either an express or implied licence. Frequently, the terms and conditions of software licences include clauses prohibiting the alteration of the software. Equally, where the hardware is subject to a patent, restrictions apply. Although a person who acquires a patented product might, for instance, repair and modify the product, they might not be permitted to modify it to the extent that they make the product anew (thereby, interfering with the patent holder’s exclusive rights over the product).^140^

IPR restrictions thus raise a number of as yet unanswered questions for the law. There are potential implications, in particular, for biohackers such as Dana discussed in the previous section. For instance, what ought the situation to be if a person alters the software in their device, thereby undertaking a restricted act and potentially infringing copyright? What if they modify the device by installing different software, replacing the original software with some that not been approved by the manufacturer (so-called jailbreaking)?^141^ One practice undertaken by manufacturers of e-devices when uses contravene the terms and conditions is to ‘brick’ devices (render them non-functional, i.e. as useful as a brick).^142^ Alternatively, they can withdraw technical support or refuse to issue updates for software so that a device can no longer function effectively.^143^ Such moves would be concerning to say the least in the context of prostheses and implanted medical devices. Withdrawing support or updates for medical devices could have very real (and potentially fatal) consequences for patients. Whilst remotely deactivating e-devices might be legally permissible ordinarily, in the everyday cyborg context, we cannot just assume that the usual entitlements of manufacturers and IPR holders pertain (or at least are enforceable).^144^

Running through the kinds of challenges and difficulties just set out is the more fundamental issue of the appropriateness of third-party control over devices, which, in essence, become integrated with persons. In such cases, (at least unconsented) interferences would seem to constitute a violation of everyday cyborg bodily integrity and the control persons are usually granted over their bodies and lives. Yet again, this throws the conceptual foundations of the law into stark relief, highlighting the challenge to the subject–object divide and law’s boundary-work that everyday cyborgs pose.

## IV. EVERYDAY CYBORGS & LAW’S BOUNDARY-WORK: CONCEPTUAL AND NORMATIVE CHALLENGES

As we noted near the beginning of this article, in general, the types of challenges we have indicated arise because of: (i) the transgression of the bodily boundary, (ii) the integration of the technologies with persons, (iii) the linking of the biological with synthetic materialities, and (iv) the nature of the technologies as integrated goods. The conceptual problem at the root of much of this is that the law considers that something is either a person (subject) or a thing (object). In this respect, the law engages in clear boundary-work; that is, it constructs a bright line boundary between person and thing which is foundational to its entire approach. Consequently, this subject–object dichotomy determines law’s structure, as well as practical matters such as the offences and remedial routes available. Throughout this article we have seen how this binary plays out in relation to a number of everyday cyborg-related challenges. We have also seen the pressure that the assemblage of integrated persons and integrated goods puts on such divisions. First, it is evident in the relevant medical device law and regulation, where it is clear that these are constructed as law’s ‘objects’. Secondly, the dichotomy was apparent when considering how the law could, or ought to, deal with damage to AIMDs. Thirdly, in relation to data, we saw that the increasing sophistication of medical devices and their interactions/integration with persons call into question divides which could be drawn between data types (something that is sometimes used to justify differential control over such data). This aspect was again highlighted in relation to biohacking where we note that the liabilities which flow from any harm done would likely follow either subject- or object-focused pathways (as distinguished from alternative or hybrid solutions).

Before drawing this article to a close, it is worth examining the subject–object dichotomy further. In so doing, we posit that the subject–object dichotomy closely parallels at least two other dichotomies for the law; specifically ones which draw lines between the internal and external and the biological and synthetic. These divisions highlight the challenges which everyday cyborgs and their technologies pose to existing legal ontologies (perceived realities which get built into law’s structure and operation)^145^ and thus to law’s boundary-work. By boundary-work, we mean how the law utilises and incorporates supposed ontological and moral boundaries, something which influences not only its approach to specific problems, but also its underlying structure. To illustrate, consider the transgression of the bodily boundary represented by much everyday cyborg technology. This is crucially important in terms of understanding the challenges for the law. The bodily boundary seemingly marks out the person from the external world and, as such, it is often the mode by which the subject–object dichotomy is given effect in particular laws (e.g. damage to property versus assault and battery). As one of us has argued elsewhere, the subject–object boundary is taken to be an ontological one (i.e. an empirical reality). This is then imbued with a moral significance that we find reflected in law’s structure and operative rules.^146^

Our use of ‘boundary-work’ here draws on discussions from science and technology studies. Originally, Gieryn employed the term to draw attention to and analyse how experts, organisations, and other actors delineate science from non-science.^147^ In so doing, they cross, erect, or erase boundaries in order to legitimise their knowledge and claims or to delegitimise the knowledge and claims of others.^148^ Boundary-work has also been used to discuss how scientists engage in boundary-work which is ‘about drawing boundaries between what is ethically preferable’.^149^ On the whole where the concept has been employed in legal scholarship, it has been done with this focus on the science/non-science boundary and how science is used as a legitimator for (lack of) regulatory activity in particular areas.^150^ Recently, it has also been used to examine how the idea of liminality (the spaces ‘in-between’) can address the problems wrought by different kinds of boundary-work in health research regulation.^151^ As Taylor-Alexander and colleagues note in their exploration of this, for law and regulation sometimes ‘a focus on margins is not very helpful’, especially in situations where boundaries move and change.^152^ This is something that comes to the fore in a very literal sense in the everyday cyborg context.

Our usage of the term to reveal and analyse challenges regarding the law and everyday cyborgs looks beyond discussions of the borders between science and non-science. This is important, because there are a range of ontological, epistemic, and moral claims which shape the law and get built into its structure—often becoming accepted as immutable realities. In this respect, our usage has resonances both with Brownsword and Goodwin’s discussion of ‘boundary-marking concepts’ and with Faulkner’s analysis of boundaries between (types of) medical devices and between the materials they are composed of. For Brownsword and Goodwin ‘boundary-marking concepts’ indicates how ideas such as human dignity, harm, equality, and so on influence people’s decision-making regarding whether or not the regulation of particular technologies is legitimate.^153^ Meanwhile Faulkner’s focus on boundaries between types of devices (and their materials) demonstrates how these are utilised to (re)draw legal, regulatory, and governance boundaries.^154^

Utilising the concept of boundary-work more broadly draws our attention to a wide variety of (types of) claims and prompts us to unpick the claims themselves and the process of how they came to be embedded in law.^155^ Importantly, it can also help us to understand what *matters* for the law. For instance, it is clear that location matters for the law; that is, the question of internal versus external. At first glance, the internal-external axis seems like an adequate and pragmatic division around which the law gives effect to via its various rules. However, there are a couple of difficulties with this. First, the bodily boundary itself, and hence the subject–object boundary, is not fixed in the biotechnological world. The everyday cyborg is a stark manifestation of this. Indeed a brief survey of devices quickly reveals the lack of fixity that exists with regard to location and the bodily boundary. Devices might be completely external (e.g. simple limb prostheses) or completely internal (e.g. knee replacements or internal pacemakers). They may also occupy some sort of intermediate category and be partially internal and partially external, such as is the case with external pacemakers. With these, the pacemaker itself is located outside of the body, but the pacing wires (through which the device’s electrical impulses are delivered to regulate the heart’s rhythm) penetrate the skin and travel through the blood vessels to the heart chambers.^156^ Moreover, what becomes internal may once again become external; for instance, a faulty device that needs to be replaced. The bodily boundary is thus unmade and remade as objects breach the skin and pass in and out of the body.

As Taylor has observed, there is a ‘tendency to presume, rather than ask, what a body is and where its significant boundaries are located’.^157^ This presumptive tendency is evident in the boundaries constructed within the law. And it is this which leads directly to the second difficulty. For the law, problems arise when that which was once external becomes (at least partially) internal.^158^ Whilst the law is structurally divided into branches relating to persons and branches relating to things, as put by Brownsword, Scotford, and Yeung, ‘technological innovation … puts pressure on traditional legal concepts (of “property”, “patentability”, “consent”, and so on)’.^159^ This is no less true of everyday cyborgs. This was particularly clear in the discussion around damage and liability earlier in the article. Here we saw that law’s need to categorise (e.g. as property or personal injury) fixes the ‘things’ at issue as either subjects or objects. The consequence of this is that law’s structure and underlying conceptual framework is challenged by everyday cyborg technologies.

However, the challenge which devices present in this respect is not simply about location (internal-external) or the (transgression of the) bodily boundary crudely understood. Crucially, it is about the level of integration with or dependency of persons in relation to devices. As stated earlier everyday cyborgs *are* integrated persons. They are the literal integration of persons and things. Devices become incorporated into persons along multiple dimensions, including the physical, functional, psychological (becoming part of their lives and identities), and phenomenological (becoming part of how they live and experience the device and the world).^160^ Of these, the most direct pressure on the law and law’s foundations comes from the physical and functional aspects of integration/dependence.

Parts and devices become physically incorporated into or integrated with persons to greater or lesser degrees. Some such as simple prosthetic devices and some wearable technologies are straightforwardly attached to persons. The bodily skin is not broken, although they do become extensions of the body itself; for instance, an aesthetic arm prosthesis. Other wearables such as external insulin pumps more explicitly transgress the bodily boundary. These pumps have a needle that is inserted under the skin. Meanwhile, hip and knee replacements, as well as implanted devices such as ICDs or pacemakers, are completely internalised. These have a higher level of physical integration with persons. For some devices this also brings with it a higher level of dependency of persons on the device. This relates to functional integration. Some devices have a mere aesthetic function, such as simple limb prostheses. Others have a simple mechanical one. Joint replacements, for example, are aimed at mimicking the movement and function of the natural joint. Yet other devices can be seen in relation to some sort of monitoring, regulatory, or interventionist functioning; or indeed some combination of these—both internal and externally worn insulin pumps monitor a person’s blood glucose, analysing and then delivering their insulin requirements.

Thus, the greater the physical integration with persons, the greater support or replacement of bodily functioning, or the greater the dependency of persons on the devices, the more the subject–object dichotomy blurs and breaks down. Significantly, any such division is completely eliminated in the case of devices which keep persons alive. Pacemakers, for example, continually monitor and regulate a person’s heart rhythm. ICDs do this too, but can also deliver an electric shock if needed. These devices can literally be the difference between life and death. As such, they become constitutive of the everyday cyborg’s subjecthood.^161^

The blurring of the subject–object dichotomy maps closely onto another divide—that between the biological and the artificial. Often the biological is more closely associated with subjects and the synthetic with objects. Consider again the discussion in Section III of the construction medical devices as law’s objects. There we argued that various regulatory and legal instruments construct medical devices as different types of ‘objects’—risk objects, marketised objects, innovation objects, and so on. Arguably, underlying all of these is an understanding of these objects as artificial, and, as such, set apart from the biological. Here we can view materials and materiality as being significant for understanding the law’s approach. This is underscored if we think about the simple fact that we have different legislation governing the synthetic and the biological—the Medical Devices Regulations 2002 (and now the MDR and IVDR) for artificial devices versus the Human Tissue Acts,^162^ Human Transplantation (Wales) Act 2013, and the Human Fertilisation and Embryology Act 1990 (as amended 2008) for various biomaterials. Even within the category of ‘biological’ we can see there is marked boundary-work taking place, most noticeably that which delineates reproductive from other tissues. Thus, for the law it is clear that the types of materials at issue matter. But it is not just that ‘matter matters’;^163^ that is, whether it is important that the materials themselves are biological or synthetic. The process of mattering—how material comes to matter—is significant.^164^

Sørensen observes that materiality is a frequent preoccupation of science and technology studies, yet it is rarely defined.^165^ Some use it to refer ‘to the “physical” aspects of entities or simply to anything “non-human”’.^166^ For others, materiality is relational, about the interrelationship between the material and the social.^167^ Influenced by this latter understanding, we use it here to draw attention to relationality and process regarding everyday cyborgs and the law.^168^ Hence, when we say that challenges arise because of the linking of the biological with synthetic materialities, we do so to highlight not only that materials (i.e. biological or synthetic) matter for law and law’s approach, but this mattering occurs as part of particular contexts, processes, and relations. For everyday cyborg technologies, and indeed everyday cyborgs themselves, the interrelationship is not only between the materials and the social, but also the legal, the conceptual, and the normative. It is through these that the everyday cyborg and their technologies come to ‘matter’ to the law, and in virtue of which the law takes one approach rather than another. For the law, the question of what matters (and how) when it comes to technologies is one which traditionally delineates persons from things along multiple dimensions (subject–object, internal–external, biological–synthetic). Yet the everyday cyborg’s very existence puts pressure on law’s boundary-work here, being—as they are—the living embodiment of the linking of the biological with synthetic–technological materialities.

When we talk about materialities (as opposed to simply materials), it serves to remind us that a number of the challenges we have pointed to in this article arise not in virtue of material objects, but because of the immaterial. The immaterial can be understood in terms of contexts, processes, and relations as just mentioned, but also in terms of the immaterial (aspects of) technologies (software, data, and wireless communication). The discussion at various points in Section III highlighted the fact that the law does not have an integrated approach for dealing with integrated goods, let alone the further integration of these with persons; for instance, how to think about IPRs once their ‘object’ is in the body, or whether data on device functioning ought to be treated all that differently from physiological data about organ functioning (e.g. heart versus pacemaker) in such circumstances. Here again maintaining an object-focused law, and thus simply applying existing law regarding non-person integrated technologies, may not be satisfactory. It may lead us to neglect more subject-orientated concerns, such as issues of process and transformation highlighted in Section III, or issues of third-party bodily control where IPRs remain with their originator. If this is correct, then we may need to find ways to surmount yet another boundary which the law gives life to: that between the material and immaterial.

## V. CONCLUSION

A key challenge, as noted in Halsbury’s Laws of England in relation to computer systems generally, is that ‘a legal system focusing on issues of ownership, control and use of physical objects must re-orientate itself to suit the requirements of an information society and the new worlds of cyberspace’.^169^ The rise of the everyday cyborg means that the law must re-orientate itself yet again. It must not only meet the needs of the new world of cyberspace, but those of the assemblage of integrated persons and integrated goods. Everyday cyborg technologies can be viewed as disruptive, not only to the practical approach of the law, but with respect to its conceptual and normative underpinnings.^170^ Given this, unless the law evolves, it is going to struggle to adequately and justifiably deal with the challenges wrought by advancing technology generally, and those of the assemblage of integrated persons and integrated good in particular. Exactly what the shape of the law should be in this respect remains to be explored and debated. Nevertheless, it is only by confronting everyday cyborgs and their technologies head on that we can better understand the challenges for the law and, more importantly, begin to imagine alternative legal futures for dealing with them.

## Footnotes

1 US Food & Drug Administration, ‘Medical Devices: Implants and Prosthetics’, available at <https://www.fda.gov/MedicalDevices/ProductsandMedicalProcedures/ImplantsandProsthetics/> accessed 2 October 2017.

2 C Camara, P Peris-Lopez and JE Tapiador, ‘Security and Privacy Issues in Implantable Medical Devices: A Comprehensive Survey’ (2015) 55 Journal of Biomedical Informatics 272, 272.

3 G Haddow and others, ‘Cyborgs in the Everyday: Masculinity and Biosensing Prostate Cancer’ (2015) 24 Science as Culture 484, 486.

4 D Haraway, ‘A Cyborg Manifesto: Science, Technology, and Socialist-Feminism in the late Twentieth Century’ in *Simians, Cyborgs and Women: The Reinvention of Nature* (Routledge 1991) 149–81, 149. The imagery of the cyborg has long been used in the sociology and science and technology studies literatures to draw our attention to and to challenge boundaries. Famously, Haraway argues that the cyborg represents a breach in the boundaries between human and animal, animal-human and machine, and the physical and non-physical (151–54). However, note that Haraway’s cyborg was a specific mythologic construction aimed at being a commentary on identity and radical feminism (amongst other things). Although we nod to her—in particular, with regard to the transgression of boundaries—our purpose is different and much less metaphoric.

5 G Haddow, SHE Harmon and L Gilman, ‘Implantable Smart Technologies (IST): Defining the “Sting” in Data and Device’ (2016) 24 (3) Health Care Analysis 210, 212.

6 ibid; Haddow and others (n 3). For the original discussion of cybernetic organisms, see ME Clynes and ND Kline, ‘Cyborgs and Space’ (1960) 5 (9) Astronautics 26.

7 Haddow and others, ibid 490. See also Haddow, Harmon and Gilman (n 5).

8 For a brief discussion of this inconsistency, see S Thomas, ‘Security Interests in IP: Proposals for Reform’ (2017) 37 Legal Studies 214, 217–18.

9 Haddow and others (n 3) 218.

10 Cf. Hildrebrand who discusses different levels of ‘smartness’ and defines these in terms of agency (in both the philosophical and computer science sense of that term). M Hildebrand, *Smart Technologies and the End(s) of Law* (Edward Elgar 2015) 21–40. The next generation of AIMDs may also include those with artificial intelligence (AI) capabilities such as utilising machine learning or natural language processing algorithms (F Jiang and others, ‘Artificial Intelligence in Healthcare: Past, Present and Future’ (2017) 2 Stroke and Vascular Neurology e000101); for instance, wearable devices which can collect and analyse data for stroke prediction (A Mannini and others, ‘A Machine Learning Framework for Gait Classification Using Inertial Sensors: Application to Elderly, Post-Stroke and Huntington’s Disease Patients’ (2016) 16 Sensors 134).

11 Conceivably, the term could also be used to encompass persons who use technologies, such as mobility assistance devices (MAD). For a case report on damage to a MAD, see L MacDonald Glenn, ‘Case study: Ethical and Legal Issues in Human Machine Mergers (or the Cyborgs Cometh)’ (2012) 21 Annals of Health Law 175. In this MacDonald Glenn raises the question of the blurring of bodily boundaries, which is a thread that runs throughout this article (177–78).

12 W Burleson and S Carrara, ‘Introduction’ in W Burleson and S Carrara (eds), *Security and Privacy for Implantable Medical Devices* (Springer Science+Business Media 2014) 1–11, 4–5.

13 G Laurie, ‘Liminality and the Limits of Law in Health Research Regulation’ (2017) 25 Medical Law Review 47, 49.

14 ibid 48–49.

15 See generally, ibid 51.

16 ibid 18.

17 Our thanks to Elen Stokes for prompting us to consider an expanded set of ways in which the law constructs ‘objects’.

18 s 2(1).

19 We say direct influence, because even if they do not become an explicit part of UK law, the UK will need to revise the law in this area if they are to continue to trade with the EU with regard to medical devices. For example, they will need to ensure compliance with EU certification, compliance, and monitoring standards. Indeed, current guidance on the Government website suggests the UK is proceeding as if these will be part of UK law. See ‘Medical devices: EU regulations for MDR and IVDR’, <https://www.gov.uk/guidance/medical-devices-eu-regulations-for-mdr-and-ivdr> accessed 2 October 2017.

20 For the implementation timetable of the new Regulations, see European Commission, ‘Regulatory Framework’, <https://ec.europa.eu/growth/sectors/medical-devices/regulatory-framework_en> accessed 2 October 2017.

21 ibid.

22 D Cohen, ‘Out of Joint: The Story of the ASR’ (2011) 342 British Medical Journal d2905.

23 Medicines and Healthcare products Regulatory Agency, ‘Medical Device Alert: All Metal-On-Metal (MoM) Hip Replacements’ (25 June 2012) <https://mhra.filecamcom/public/file/2n8j-aq8bkp2l> accessed 2 October 2017.

24 Tens of thousands of patients have these types of joints and in June 2017, the MHRA issued guidance on follow-up and patient management to ensure the early detection of any problems. Medicines and Healthcare products Regulatory Agency ‘Medical Device Alert: All metal-on-metal (MoM) hip replacements: updated advice for follow-up of patients’ (29 June 2017) <https://www.gov.uk/drug-device-alerts/all-metal-on-metal-mom-hip-replacements-updated-advice-for-follow-up-of-patients> accessed 2 October 2017. Litigation involving over 600 individual claimants is set to go ahead from October 2017 in what law firm Leigh Day are calling ‘one of the largest product liability group actions in recent years’. Leigh Day, ‘Metal-on-metal hip trial set for October 2017’ (7 July 2016), <https://www.leighday.co.uk/News/News-2016/July-2016/Metal-on-Metal-Hip-Trial-Set-for-October-2017> accessed 2 October 2017.

25 Medicines and Healthcare products Regulatory Agency, ‘Medical Device Alert: Silicone Gel Filled Breast Implants Manufactured by Poly Implant Prothese (PIP) - All Models and Lot Numbers’ (31 March 2010), <https://www.gov.uk/drug-device-alerts/medical-device-alert-all-models-and-lot-numbers-of-silicone-gel-filled-breast-implants-unapproved-composition-of-silicone-gel> accessed 2 October 2017.

26 Department of Health, *Poly Implant Prothèse (PIP) Breast Implants: Final Report of the Expert Group* (Department of Health, June 2012) <https://www.gov.uk/government/uploads/system/uploads/attachment_data/file/214975/dh_134657.pdf> accessed 2 October 2017.

27 European Commission, ‘New EU Rules to Ensure Safety of Medical Devices’, <http://europa.eu/rapid/press-release_MEMO-17-848_en.htm> accessed 2 October 2017.

28 S Hilgartner, The Social Construction of Risk Objects: Or How to Pry Open Networks of Risk’ in SF James and LWW Clarke (eds), *Organizations, Uncertainties, and Risk* (Westview Press 1992) 39–53, 41.  

29 Annex I.I Directive 93/42/EEC.

30 Annex I.I Directive 90/385/EEC.

31 See Part II of the Consumer Protection Act 1987.

32 ibid, s 19(1)(b).

33 The MDR and IVDR are voluminous pieces of legislation. For summary of various aspects of them see the three-part overview of the Regulations from Squire Patton Boggs: Part I, <http://www.squirepattonboggs.com/∼/media/files/insights/publications/2017/01/eu-medical-device-and-ivd-regulations-overview-series-part-1/25810–eu-medical-device-alert.pdf> accessed 2 October 2017; Part II, <http://www.squirepattonboggs.com/∼/media/files/insights/publications/2017/02/eu-medical-device-regulation-overview-part-2/25829life-scienceseu-medical-device-regulation-overview-part-2thought.pdf> accessed 2 October 2017; and Part III <http://www.squirepattonboggs.com/∼/media/files/insights/publications/2017/02/eu-in-vitro-diagnostic-medical-device-regulation-overview-part-3/25830life-scienceseu-in-vitro-diagnostic-medical-device-regulation-ove.pdf.> accessed 2 October 2017.

34 Recitals 1 and 2.

35 MDR, Recital 59 and Chapter V; and IVDR, Recital 55 and Chapter V.

36 MDR, Recital 63 and Chapter VI; and IVDR, Recital 61 and Chapter VI.

37 MDR, Recital 74 and Chapter VII; and IVDR, Recital 75 and Chapter VII.

38 ML Flear, ‘Regulating New Technologies: EU Internal Market Law, Risk, and Socio-Technical Order’ in M Cremona (ed), *New Technologies and EU Law* (OUP 2016) 74–122, 77.

39 ibid, 80–83.

40 Article 168 Treaty on the Functioning of the European Union on public health. Our thanks to Daithí Mac Síthigh and Mark Flear for drawing our attention to this point.

41 See European Commission, ‘Innovation Union’ <http://ec.europa.eu/research/innovation-union/index_en.cfm> accessed 2 October 2017.

42 See European Commission, ‘Public Health: Europe 2020 – for a Healthier EU’ <https://ec.europa.eu/health/europe_2020_en> accessed 2 October 2017. For an in-depth and nuanced case regarding the link between innovation, health, and the EU internal market, see Flear (n 38).

43 See generally the text of the AIMDD, MDD, IVD, 2002 UK Regulations, MDR, and IVDR.

44 Flear (n 38) 115–17.

45 E Stokes, ‘Recombinant Regulation: EU Executive Power and Expertise in Responding to Synthetic Biology’ in *Regulating Risks in the European Union: The Co-production of Expert and Executive Power* (Bloomsbury Publishing 2017) 59–79, 64.

46 See, for example, the European Commission landing page on the medical device sector <https://ec.europa.eu/growth/sectors/medical-devices_en> accessed 2 October 2017. Moreover, note how the header on this page is ‘Growth: Internal Market, Industry, Entrepreneurship, and SMEs’.

47 Stokes (n 45) 79.

48 This same wording is found in Recitals 1 and 2 of both the MDR and IVDR.

49 Our thanks to Aisling McMahon for this point.

50 Laurie (n 13) 51. Whilst Laurie’s discussion centres on health research regulation, his observations are much more broadly applicable vis-à-vis health and health technology regulation.

51 M Quigley, *Self-ownership, Property Rights, and the Human Body: A Legal and Philosophical Analysis* (CUP 2018) 272.

52 Laurie (n 13) 61 [emphasis in original].

53 ibid 62.

54 We thank one of the anonymous reviewers for their thoughts on risk and feedback loops.

55 Hilgartner (n 28) 42.

56 K Hoeyer, *Exchanging Human Bodily Material: Rethinking Bodies and Markets* (Springer 2013), vi.

57 See Laurie (n 13) for process and transformation in health research regulation.

58 ibid 68.

59 Quigley (n 51) 253.

60 Department of Health and Social Security, ‘Health Services Management Ownership of Implants and Removal of Cardiac Pacemakers After Death’ Health Notice HN(83)6 (London 1983) <http://webarchive.nationalarchives.gov.uk/20141206201515/http://www.mhra.gov.uk/home/groups/dts-bi/documents/websiteresources/con123256.pdf> accessed 2 Oct 2017.

61 See MHRA, ‘Leaving Hospital With a Medical Device’ <http://webarchive.nationalarchives.gov.uk/20130513170749/http://www.mhra.gov.uk/Safetyinformation/Generalsafetyinformationandadvice/Adviceandinformationforconsumers/Leavinghospitalwithamedicaldevice/index.htm> accessed 2 October 2017.

62 See, for example, advice from Bevan Brittan and Clark Wilmott, both of which reference the notice <https://www.bevanbrittan.com/insights/articles/2012/metalonmetalhipreplacements> accessed 2 October 2017 and <https://www.clarkewillmott.com/blog/ownership-of-medical-implants/> accessed 2 October 2017.

63 D Dickenson, *Property in the Body: Feminist Perspectives* (2nd edn, CUP 2017) 5. See also Quigley (n 51), ch 8, s 3.

64 Although we note that fundamentally all law regulates dealings between persons (or legal persons such as corporations), even law which is about objects.

65
*R v Bentham* [2005] UKHL 18 [1].

66 ibid [8].

67 ibid.

68 M Quigley, ‘Property in Human Biomaterials: Separating Persons and Things?’ (2012) 32 Oxford Journal of Legal Studies 659.

69 I Goold, H Maslen and C Auckland, ‘Damage to Prostheses and Compensation for Harm’, (2017) Working Paper, 19.

70 ibid.

71 ibid 14.

72 ibid 15.

73 What follows is a necessarily brief indication of the issues involved for both the criminal and civil. For an in-depth discussion of these, including a fulsome one of remedies and penalties, see Goold, Maslen, and Auckland’s informative article, ibid.

74 Sentencing Guidelines Council, *Magistrates’ Court Sentencing Guidelines: Definitive Guideline* (Crown Copyright 2008) 44.

75 Sentencing Council, *Assault: Definitive Guideline* (Crown Copyright 2011) 24.

76 Sentencing Guidelines Council (n 74).

77 Sentencing Council (n 75) 12.

78 For a general discussion of damages in these areas, see A Burrows, *Remedies for Torts and Breach of Contract* (3rd edn, OUP 2004), chs9 and 10, and H McGregor, *McGregor on Damages* (Sweet and Maxwell 2014), especially chs 4, 35, 36, and 38. For specific discussion in the case of prostheses, see Goold, Maslen and Auckland (n 69).

79 See, eg *The London Corporation* [1935] P 70 at 77 and *Coles v Hetherton* [2013] EWCA Civ 1704 [28], [2014] 3 All ER 377 [28] p.

80 See, eg, *The Columbus* (1849) 3 Wm Rob 158 at 162 and *The Maersk Colombo* [2001] 2 Lloyd's Rep 275.

81
*O’ Grady v Westminster Scaffolding Ltd* [1962] 2 Lloyd’s Rep 238.

82 See, eg *The Hebridean Coast* [1960] 2 All ER 85 at 94, [1960] 3 WLR 29 at 40–41 and *The Naxos* [1972] 1 Lloyd's Rep 149.

83
*British Transport Commission v Gourley* [1956] AC 185; *Livingstone v Rawyards Coal Co* (1880) 5 App Cas 25 39.

84 As stated by McGregor, ‘no authority is needed to support this statement, for cases are legion which include such outlays in the damages awarded’. McGregor (n 78) 38–181.

85
*George v Pinnock* [1973] 1 WLR 118 at 124H-124C.

86 This is done in accordance with guidelines set out by the Judicial College. Judicial College, *Guidelines for the Assessment of General Damages in Personal Injury Cases* (14th edn, OUP 2017).

87 Laurie (n 13) 71.

88 ibid.

89 S Thomas, ‘Sale of Goods and Intellectual Property: Problems with Ownership’ (2014) 96 Intellectual Property Forum 25, 25.

90 [1996] EWCA Civ 1296.

91 See, for example, National Institute for Health and Care Excellence, ‘CareLink Network Service for Remote Monitoring of People with Cardiac Devices’ Medtech Innovation Briefing (24 May 2016) 1–5, <https://www.nice.org.uk/advice/mib64> accessed 2 Oct 2017.

92 K Fu, ‘Inside Risks - Reducing Risks of Implantable Medical Devices: A Prescription to Improve Security and Privacy of Pervasive Health Care’ (2009) 52(6) Communications of the ACM 25, 26; Camara, Peris-Lopez and Tapiador (n 2); H Burri and D Senouf, ‘Remote Monitoring and Follow-Up of Pacemakers and Implantable Cardioverter Defibrillators’ (2009) 11 Europace 701, 701.

93 For a small sample, see L Stevens, C Dobbs, KH Jones and G Laurie, ‘Dangers from Within?: Looking Inwards at the Role of Maladministration as the Leading Cause of Health Data Breaches in the UK’ in R Leenes and others (eds), *Data Protection and Privacy: (In)visibilities and Infrastructures. Law, Governance and Technology Series* (Springer 2017) 1–29; G Laurie and others, A *Review of Evidence Relating to Harm Resulting from Uses of Health and Biomedical Data* (Nuffield Council on Bioethics 2015); MJ Taylor, ‘Health Research, Data Protection, and the Public Interest in Notification’ (2011) 19 Medical Law Review 267; B Prainsack, ‘Genetic Data and the Law: A Critical Perspective on Privacy Protection’ (2014) 22 Medical Law Review 291; and 11.JM Victor, ‘The EU General Data Protection Regulation: Toward a Property Regime for Protecting Data Privacy’ (2013) 123 Yale Law Journal 513.

94 L Blake, ‘Health Care in the Digital Age: Who Owns the Data?’ *Wall Street Journal* (28 November 2012) <http://live.wsj.com/video/health-care-in-the-digital-agewho-owns-the-data/28B6E0AD-8506-40B2-A659-20A9B696F524.html#!28B6E0AD-8506-40B2-A659-20A9B696F524> accessed 2 October 2017.

95 ibid.

96 ibid.

97 ibid.

98 ibid.

99 For an overview of the GDPR see Information Commissioner’s Office, ‘Overview of the General Data Protection Regulation’ (17 August 2017), available at <https://ico.org.uk/media/for-organisations/data-protection-reform/overview-of-the-gdpr-1-12.pdf> accessed 2 October 2017.

100 s 1(1)(e).

101 s 2. While the type of data discussed in this article would by its nature be quintessentially personal data, there have been cases where it has not been so clear whether data would constitute personal data. For an example of these cases, see *Department of Health v Information Commissioner* [2011] EWHC 1430 (Admin) (Cranston J); *R (on the application of W, X, Y and ) v Secretary of State for Health v The Secretary of State for the Home Department* [2015] EWCA Civ 1034, (Lord Dyson MR).

102 s 2(2).

103 Recital 30.

104 GDPR, art 9 and DPB, s 33(4)-(5).

105 s 33(8). Definitions of these types of data is provided in s 184.

106 GDPR, Recitals 32 and 40, and arts 6 and 7.

107 GDPR 9(2)(a). Although see 9(2)(h) and (i) for potential health-related exemptions.

108 DPA 1998, s 7, and schs 2 and 3; GDPR. Recital 63; and DPB, s 43.

109 GDPR, Recital 68 and art 20.

110 See, for example, GDPR, art 89.

111 DPA, schs 3 and 8 and GDPR, Recitals 50-54 and art 9.

112 GDPR, art 5(1)(c).

113 One particular issue of interest is the requirement, under certain circumstances, for data protection by ‘design and by default’—in other words ‘privacy by design’—to be built into a range of technical, systems, and organisational measures (GDPR, Recital 78 and art 25). These include designing in security measures and data minimisation. For a brief discussion of this, see Information Commissioner’s Office, *Big Data, Artificial Intelligence, Machine Learning, and Data Protection* (2017) 72–74, <https://ico.org.uk/media/for-organisations/documents/2013559/big-data-ai-ml-and-data-protection.pdf> accessed 2 October 2017.

114 Our thanks to one of the anonymous reviewers for this.

115 BBC Radio 4, ‘Meet the Cyborgs: Five People Who Have Modified Their Bodies with Tech’, <http://www.bbc.co.uk/programmes/articles/1g2gNvk4Gc4MwYqhtQ8KNlf/meet-the-cyborgs-five-people-who-have-modified-their-bodies-with-tech> accessed 2 October 2017.

116 ‘The #WeAreNotWaiting Movement is Helping People with Diabetes Improve Their Health Now, Not Later’, <https://www.diabetes.co.uk/blog/2016/07/the-wearenotwaiting-movement-is-helping-people-with-diabetes-improve-their-health-now-not-later/> accessed 2 October 2017.

117 BBC Radio 4 (n 115).

118 ibid. Some of the platforms that have been launched as part of the movement include OpenAps.org (which was founded by Dana), Nightscout, x-Drip, and the Tidepool; See ‘The #WeAreNotWaiting Movement’ (n116).

119 US Government of Accountability Office, ‘Medical Devices: FDA Should Expand Its Consideration of Information Security for Certain Types of Devices’ (US Government of Accountability Office, 31 August 2012) (GAO-12-816)15, <http://www.gao.gov/products/GAO-12-816> accessed 2 October 2017.

120 See K McGowan, ‘I Want to Know What Code is Running Inside My Body’ *Wired* (2 November 2016) <https://www.wired.com/2016/02/i-want-to-know-what-code-is-running-inside-my-body/> accessed 2 October 2017; L Tung, ‘Marie’s Mission to Highlight Flaws in Medical Devices’, *Sunday Morning Herald* (27 November 2015), <http://www.smh.com.au/it-pro/security-it/maries-mission-to-highlight-fatal-flaws-in-medical-devices-20151126-gl9dwp.html> accessed 2 October 2017; D Pauli, ‘Thousands of “Directly Hackable” Hospital Devices Exposed’, *The Register* (29 September 2015) <https://www.theregister.co.uk/2015/09/29/thousands_of_directly_hackable_hospital_devices_found_exposed/> accessed 2 October 2017; and BBC, ‘Cyber-flaw Affects 745,000 Pacemakers’ (30 August 2017) <http://www.bbc.co.uk/news/technology-41099867> accessed 2 October 2017.

121 A Peterson, ‘Yes, Terrorists Could Have Hacked Dick Cheney’s Heart’, *Washington Post* (21 October 2013) <https://www.washingtonpost.com/news/the-switch/wp/2013/10/21/yes-terrorists-could-have-hacked-dick-cheneys-heart/> accessed 2 October 2017.

122 W Barfield and A Williams, ‘Law, Cyborgs, and Technologically Enhanced Brains’ (2017) 2 Philosophies 1, 4.

123 Camara, Peris-Lopez and Tapiador (n 2) 273; K Fu, ‘Inside Risks - Reducing Risks of Implantable Medical Devices: A Prescription to Improve Security and Privacy of Pervasive Health Care’ (2009) 52 Communications of the ACM 25, 26.

124 See Associated Press, ‘Robotic Arm Man Christian Kandlbauer Dies in Hospital after Crash’ *The Guardian* (22 October 2010) <https://www.theguardian.com/world/2010/oct/22/christian-kandlbauer-arm-dies-crash> accessed 2 October 2017.

125 As well as the tort of negligence this includes and the Medical Device Regulations 2005, this includes a range of consumer protection legislation, for example, Misrepresentations Act 1967; Trade Descriptions Act 1968; Consumer Protection Act 1989 (implementing EC Directive 85/374 on Liability for Defective Products); General Product Safety Regulations 2005; Consumer Contracts (Information, Cancellation and Additional Charges) Regulations 2013, SI 2013/3134; and Consumer Rights Act 2015.

126 Barfield and Williams (n 122), 3.

127 ss 1–3.

128 ibid.

129 Thanks to one of the anonymous reviewers for prompting further thoughts on this.

130 Our thanks to Imogen Jones for her thoughts on this.

131 Note that we often refer to property in chattels as personal property, meaning property in tangible goods. However, and perhaps somewhat confusingly, IPRs can also be referred to as personal property. See the Patents Act 1977, s 30(1), the Copyright, Designs and Patents Act 1988, s 90(1), and the Trade Marks Act 1994, s 24(1). Note, however, that some argue that IP does not share enough features with other forms of property (ie either land or chattels) to be viewed as property properly so-called. See, for example, S Douglas and B MacFarlane, ‘Defining Property Rights’ in J Penner and H Smith (eds), *Philosophical Foundations of Property Law* (Oxford University Press 2013) 219–43, 239.

132 The relevant legislation is as follows: (i) the Patents Act 1977 (1977 Act), (ii) the Trade Marks Act 1994 (iii) Registered Designs Act 1949, and (iv) Copyright, Designs and Patents Act 1988.

133 Computer programs, designs for such programmes, and databases are all ‘literary works’ under the Copyright Designs and Patents Act 1988, ss 3(1)(a)-(d). See also Barfield and Williams (n 122) 6.

134 JR Carvalko, ‘Law and Policy in an Era of Cyborg Assisted-Life: The Implications of Interfacing In-The-Body Technologies to the Outer World’ (2013) *Technology and Society IEEE International Symposium* 1, 6.

135 D Bainbridge, *Intellectual Property* (9th edn, Pearson Education Ltd 2012) 12; Carvalko (n 134) 6.

136 For an in-depth discussion of the modes of transfer for different types of IPRs, see M Smith and N Leslie, *The Law of Assignment* (2nd edn, OUP 2013) 451–70.

137 Patents Act 1977, s 30, Copyright, Designs and Patents Act 1988, s 90, and Trade Marks Act 1994, s 24.

138
*Cooper v Stephens* [1895] 1 Ch 567. For the formalities relating to an assignment of copyright, see Copyright, Designs and Patents Act 1988, s 90 (3); For the formalities relating to assignment of a patent, see Patents Act 1977, s 30 (6). See also L Bently and B Sherman, *Intellectual Property Law* (4th edn, OUP 2014) 294.

139 See Bently and Sherman, ibid 644.

140 See *Schutz v Werit* [2013] UKSC 16; *United Wire v Screen Repair Services* [2000] 4 All ER 353 (HL), 358 (Lord Hoffman); *British Leyland v Armstrong* [1986] RPC 279, 376.

141 See G Ramachandran, ‘Assault and Battery on Property’ (2015) 44 Loyola of Los Angeles Law Review 253, 256–57. For a brief discussion of jail-breaking with regard to smartphones, see D Mac Síthigh, ‘App Law Within: Rights and Regulation in the Smartphone Age’ (2013) 21 International Journal of Law and Information Technology 154, 168–70.

142 See, for example, G Corfield ‘DJI's Spark Drones to be Bricked by September 1 Unless Firmware Updated’ *The Register* (22 August 2017) <https://www.theregister.co.uk/2017/08/22/dji_spark_brick_threat_1_sep_firmware_update/> accessed 2 October 2017 and K Collins, ‘HP is Now Letting Consumers Remove Software that Blocks Third-party Ink Cartridges in its Printers’ (30 September 2016) <https://qz.com/797489/hp-was-blocking-third-party-ink-cartridges-in-its-printers-but-now-is-relenting/> accessed 2 October 2017.

143 See Ramachandran (n 141). Although dealing with non-medical wearable devices, see also Google, ‘Glass Terms of Use’ (19 August 2014) <https://www.google.com/glass/termsofuse/> accessed 2 October 2017. Note the provisions under the heading ‘Software and Services’ relating to installing unauthorised software and making alterations to the device.

144 It is worth noting that even if the IP in prostheses and implants was transferred to a purchaser by virtue of the sale of the device, this would not further the everyday cyborg’s case for ownership because the intellectual property will potentially not be assigned to the person to whom the device is attached, that is the everyday cyborg. This is because in these transactions, the purchaser of the device could be someone other than the everyday cyborg. For instance, where the device has been obtained through the National Health Service (NHS) rather than through a private sale, the transaction for the sale of the prosthesis or implant would not involve the everyday cyborg. Rather it would be between the supplier of the device and the NHS, with the NHS being the purchaser of the device. An argument could be made that the NHS would be acting as an agent on behalf of the everyday cyborg because the payment that would be made in any sale transaction will come from the public purse, and therefore from the everyday cyborg through the payment of taxes, and National Insurance contributions, if he or she is in employment. However, while there might be some merit in this argument, the point remains that the contract for the sale of the prosthesis or implant will be between the supplier of the device, and the NHS, rather than between the supplier of the device, and the everyday cyborg. Of course, this difficulty will not arise if the device is obtained through a private sale where the everyday cyborg makes a payment in exchange for the device. However, the existence of an NHS that is free at the point of delivery in the UK, and which offers a range of prostheses and implants as treatment for a number of conditions presumably means that in the UK at least many of these devices will have been, and are acquired through the NHS, rather than through private sales.

145 This use of the phrase ‘legal ontologies’ is to be distinguished from its use at the interface of law and computer science where it means ‘a vocabulary and set of rules for constructing assertions’ (D Rubin, ‘How Legal Robot Works: Legal Ontologies’ <https://www.legalrobot.com/blog/2016/05/25/Ontologies/> accessed 2 October 2017.

146 Quigley (n 51) 242–55.

147 TF Gieryn, *Cultural Boundaries of Science* (University of Chicago Press 1999) 27; TF Gieryn, ‘Boundary-Work and the Demarcation of Science from Non-Science: Strains and Interests in Professional Ideologies of Scientists’ (1983) 48 American Sociological Review 781; M Lamont and V Molnar, ‘The Study of Boundaries in the Social Sciences’ (2002) 28 Annual Review of Sociology 167.

148 Lamont and Molnar, ibid 179.

149 SP Wainwright and others, ‘Ethical Boundary-Work in the Embryonic Stem Cell Laboratory’ (2006) 28 Sociology of Health and Illness 732, 739.

150 See, for example, S Jasanoff, *The Fifth Branch: Science Advisors as Policymakers* (Harvard UP 1990).

151 S Taylor-Alexander and others, ‘Beyond Regulatory Compression: Confronting the Liminal Spaces of Health Research Regulation’ (2016) 8 Law, Innovation, and Technology 149, 170–72.

152 ibid 172.

153 R Brownsword and M Goodwin, *Law and the Technologies of the Twenty-Frist Century* (CUP 2010) 187–224.

154 A Faulkner, *Medical Technology into Healthcare and Society: A Sociology of Devices, Innovation and Governance* (Palgrave MacMillan 2009).

155 This usage of boundary-work should, however, be differentiated from both ‘boundary objects’ and ‘bounded objects’. The former are objects which mediate between different social communities, in which there may be both shared and divergent understandings of the object (SL Star and JR Griesemer, ‘Institutional Ecology, “Translations” and Boundary Objects: Amateurs and Professionals in Berkeley’s Museum of Vertebrate Zoology, 1907-39’ (1989) 19 Social Studies of Science 387; GC Bowker and SL Star, *Sorting Things Out: Classification and Its Consequences* (MIT Press 2000); SL Star, ‘This is Not a Boundary Object: Reflections on the Origin of a Concept’ (2010) 35 Science, Technology, and Human Values 601; and NJ Fox, ‘Boundary Objects, Social Meanings and the Success of New Technologies’ (2011) 45 Sociology 70). The latter has been used to describe the bounded regulatory categories that the law creates with regard to certain ‘objects’: tissue, data, embryos, and so on (see Taylor-Alexander and others (n 151) 160).

156 Other devices such as external insulin pumps could be viewed as both *wearable* and *penetrable*—these are externally worn devices, but the needles that deliver the insulin pierce the skin. As Haddow and others put it, ‘these technologies are performing not only *for us*, but also *on us* and *within us*, chipping away at the inaccessibility of our internal bodies’. Haddow, Harmon and Gilman (n 5) 211.

157 JS Taylor, ‘Surfacing the Body Interior’ (2005) 34 Annual Review of Anthropology 741, 749.

158 Analogous problems arise for biomaterials that go the other way, from the body to the external world.

159 R Brownsword, E Scotford and K Yeung, ‘Law, Regulation, and Technology: The Field, Frame, and Focal Questions’ in R Brownsword, E Scotford and K Yeung (eds), *Oxford Handbook of the Law and Regulation of Technology* (OUP 2017) 3–38, 3.

160 Quigley (n 51) 251–55.

161 ibid 252.

162 Human Tissue Act 2004 (England, Wales, and NI) and Human Tissue (Scotland) Act 2006.

163 J Law, ‘The Materials of STS’ in D Hicks and MC Beaudry (eds), *The Oxford Handbook of Material Culture Studies* (OUP 2010) 173–88, 173.

164 ibid.

165 E Sørensen, ‘The Time of Materiality’ (2007) 8, Art 2 Forum: Qualitative Social Research 3.

166 ibid.

167 ibid 4, referring to the work of Bruno Latour in particular. See B Latour, *We Have Never Been Modern* (Harvester Wheatsheaf 1993).

168 In so doing, we do not purport to adhere to any particular or strict usage of the term. Indeed, as Sørensen points out, ‘neither Latour [from whom relational/agency accounts have their antecedence] nor STS scholars in general occupied with materiality dwell on the efforts to formulate a clear definition of materiality’; Sørensen (n 165).

169
*Halsbury’s Laws of England* (2012) vol 57, para 502.

170 For an in-depth discussion of three modes of disruption posed by technology in general, see Brownsword, Scotford and Yeung (n 159)

